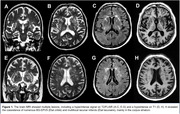# Coexistence of État criblé and État lacunaire in a patient with mixed dementia: a case report

**DOI:** 10.1002/alz70856_102071

**Published:** 2025-12-25

**Authors:** Paulo Eduardo Lahoz Fernandez, Alan Cronemberger Andrade, Paulo Henrique Ferreira Bertolucci

**Affiliations:** ^1^ Federal University of São Paulo ‐ UNIFESP, São Paulo, SP, Brazil

## Abstract

**Background:**

The Virchow Robin spaces are fluid‐filled perivascular spaces surrounding cerebral perforating arteries. Enlarged perivascular spaces (EPVS) are well‐defined round/ovoid shapes, usually present on basal ganglia (BG‐EPVS) and centrum semiovale (CSO‐EPVS). Lacunar infarcts are small lesions (< 15 mm diameter) in the distal distribution of deep penetrating vessels. These MRI lesions are associated with a higher risk for cognitive decline in vascular dementia (VaD) and Alzheimer's disease (AD), giving rise to mixed dementia (MD). État criblé is a term that describes the cribriform state seen in the MRI, characterized by symmetrical and diffusely widened punctiform EPVS often seen in the white matter (WM) and BG (corpus striatum and globus pallidus). État lacunaire describes the presence of multiple lacunar infarcts due to occlusion of penetrating cerebral arterioles, especially in the BG.

**Method:**

We report the clinical and imaging features of a case of MD, revealing a rare MRI pattern with multiple EPVS and multifocal lacunar infarcts, characterizing the coexistence of État criblé and État lacunaire.

**Result:**

An 85‐year‐old woman showed a 6‐year progressive amnestic and dysexecutive cognitive impairment. She started forgetting appointments, paying bills, showing repetitive questions with slowed processing speed, poor task planning/organization, and mild apathy, which impacted her daily activities. The neurological exam was unremarkable, and the familiar history was negative. On neuropsychological evaluation, she scored 19/30 on MMSE, 9 on semantic verbal fluency, and 5/3 on the digit span test. Brain MRI revealed temporal mesial and hippocampal atrophy (Scheltens 2). It also showed chronic WM changes (Fazekas 2), with hyperintense T2/FLAIR and hypointense T1 lesions, revealing multiple BG‐EPVS and, to a lesser extent, CSO‐EPVS. It also showed multifocal lacunar infarcts in the BG, mainly in the corpus striatum.

**Conclusion:**

It has been reported that cognitive decline is more related to BG‐EPVS than CSO‐EPV, especially when associated with lacunar infarcts, as in this case. This rare MRI pattern reinforces the importance of considering the coexistence of different subcortical lesions in BG and other deep areas in patients with MD and other dementias.